# ^1^H NMR-based metabolomics reveals metabolic changes in porcine ingesta and serum during *Ascaris suum* infection

**DOI:** 10.1186/s13071-026-07423-z

**Published:** 2026-05-23

**Authors:** Liane Wagner, Andrea Springer, Sarina Koehler, Dagmar A. Brüggemann, Christina Strube

**Affiliations:** 1 Institute for Parasitology, Centre for Infection Medicine, University of Veterinary Medicine Hannover, Buenteweg 17, 30559 Hanover, Germany; 2 Department of Safety and Quality of Meat, Max Rubner-Institut, Federal Research Institute of Nutrition and Food, E.-C.-Baumann-Straße 20, 95326 Kulmbach, Germany

**Keywords:** Nuclear magnetic resonance spectroscopy, Ascariasis, Roundworms, Pigs, Trickle-infection, Short chain fatty acids

## Abstract

**Background:**

Roundworm infections are of major importance both for humans and livestock. The porcine roundworm *Ascaris suum*, the most economically important nematode in pig production worldwide, serves as a valuable model for human ascariosis, yet knowledge of its metabolic impact remains limited.

**Methods:**

Metabolic changes were investigated in pigs infected once with 10,000 *A. suum* eggs versus trickle-infected pigs (1000 eggs/day over 10 days) compared with uninfected controls. Ingesta and serum samples of six pigs each were collected on days 21, 35, and 49 post infection (pi) for nuclear magnetic resonance (NMR)-based metabolomics analyses.

**Results:**

Trickle-infected pigs showed more pronounced metabolic changes than single-infected pigs, following a triphasic temporal pattern with initial changes at day 21 pi, maximal disruption at day 35 pi, and partial recovery by day 49 pi. The colon exhibited the most significant changes in short-chain fatty acids (SCFAs) and amino acids. On day 21 pi, trickle-infected pigs showed increased acetate, butyrate, valerate, and amino acids in the colon, with reversed patterns on day 35 pi. Serum changes mirrored colonic alterations, suggesting the colon as primary driver of systemic responses. Single-infected pigs showed less pronounced changes, with increased lactate and acetate in the ileum and elevated amino acids in the cecum on day 35 pi.

**Conclusions:**

These findings reveal complex, compartment-specific host–parasite–microbiome interactions, with SCFAs as important mediators. Enhanced growth performance in trickle-infected pigs corresponding with metabolic recovery challenges exclusively antagonistic host–parasite relationships. This study deepens the understanding of *A. suum* pathophysiology and provides crucial insights for human ascariosis, supporting targeted interventions for animal and human health.

**Graphical abstract:**

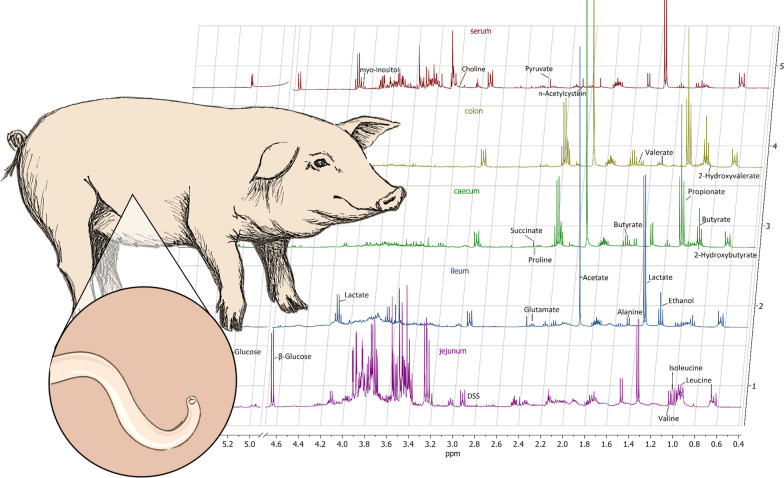

**Supplementary Information:**

The online version contains supplementary material available at 10.1186/s13071-026-07423-z.

## Background

Roundworm infections are of major importance for humans as well as in livestock farming. *Ascaris lumbricoides* is among the most important human parasites, leading to malnutrition and mental and physical underdevelopment, especially in children living in poverty in less developed countries [1]. The human roundworm *A. lumbricoides* and the zoonotic porcine roundworm *Ascaris suum* are very closely related ascarids [2], sometimes even considered as members of a single species [3]. *Ascaris suum* is the most common and economically important nematode in pig farming worldwide. Recent studies demonstrate substantial infection rates in pigs, with prevalence ranging from 26.4% on the basis of liver lesion monitoring in Italian abattoirs [4] to 48–64% in starter and finisher pigs on Danish organic farms [5] and 82% in German outdoor pig farms [6]. Economic losses are due to several factors [7], including (1) impact on growth performance as shown in reduced weight gain, feed intake, feed utilization, and digestibility of dry matter and crude protein during *A. suum* infection; (2) condemnation or downgrading of livers due to so-called milk spots as evidence of liver migratory activity of *A. suum*; and (3) potential interference with vaccinations and higher risk of coinfections, e.g., with pathogenic *E. coli*.

Infection with *Ascaris* spp. occurs through ingestion of embryonated eggs from the environment. The third-stage larvae hatch in the small intestine and penetrate the intestinal wall to reach the liver and then the lungs via the bloodstream. They reenter the intestine after being transported to the oral cavity and swallowed, which occurs approximately 8–10 days post infection (pi) in the case of *A. suum* [8]. In pigs, a so called self-cure reaction eliminates a part of the worm burden from the small intestine approximately 2–3 weeks after infection. This reaction has been associated with eosinophilia and an increase in intraepithelial T-cells in the jejunum [9]. The remaining larvae finally molt to immature worms between days 21 and 29 pi, [10], which reach patency approximately 6–8 weeks pi [11].

Roundworm infection causes profound alterations of the host’s intestinal physiology. Previous studies have shown an impairment of intestinal glucose and amino acid absorption by *A. suum* in vivo [12, 13] and by *A. suum* antigens in an in vitro setup [14]. An altered metabolic state may also result from changes in the intestinal microbiota that occur during *A. suum* infection [15–17]. Zaiss et al. [18] reported an increase of short-chain fatty acids (SCFAs), which are primarily produced by bacterial fermentation, in the porcine colon 8 weeks after oral infection with *A. suum* eggs. Due to their antiinflammatory properties, increased production of SCFAs, which has been observed in a number of helminth infections, is thought to be beneficial for both the parasites and the host [18, 19].

A comprehensive, nontargeted “fingerprint” investigation of metabolic changes during *A. suum* infection may reveal further insights into the physiological consequences of roundworm infection and potential mechanisms shaping the host–parasite relationship. Metabolomics, a promising and high-throughput tool, is defined according to Goodacre et al. [20] as the comprehensive analysis of the whole metabolome under a given set of conditions. It allows for the identification and quantification of changes of all small metabolites with a molecular weight of < 1.5 kDa, such as lipids, amino acids, carbohydrates, peptides, organic acids, vitamins, and any other chemical that can be used, ingested, or synthesized by a cell, organ, or organism [21]. To analyze a wide range of metabolites with different properties and concentrations in a single sample to assess the significance of differences and changes in the composition, a robust and reproducible analytical technique with a high capacity for high-throughput analyses is necessary. Nuclear magnetic resonance (NMR) spectroscopy fulfills these criteria and is a highly effective method in fingerprint analyses. Additionally, little-to-no sample preparation is required for the detection of a variety of metabolites simultaneously in a nondestructive, quantitative, and time-effective way. Therefore, the application of NMR-based metabolomics has gained increasing importance in recent years, especially in combination with multivariate statistical methods such as principal component analysis (PCA) and (orthogonal) partial least squares-discriminant analysis [(O)PLS-DA], independently of the studied organism [22–25].

The aim of the present study was to explore the impact of *A. suum* infections on the porcine metabolome in different intestinal compartments as well as in the bloodstream. These insights are also relevant for human health, as *A. suum* infections in pigs serve as a model for human ascariosis. As repeated low-dose infections are common under natural conditions, and may have different consequences compared with single-dose experimental infections, two infection groups, one single-infected and another trickle-infected over the course of 10 days, were compared with an uninfected control group during the early phase of *A. suum* infection.

## Methods

### Experimental design

A detailed description of the experimental design, including experimental *A. suum* infections and sample collection, can be found in Springer et al. [17]. In brief, 54 helminth-free pigs (hybrid German Landrace, 5 weeks old, weight ~10 kg) were housed in groups on straw bedding and received a standard pig diet ad libitum (Deuka Ferkelstarter Primo, Deutsche Tiernahrung Cremer, Düsseldorf, Germany). The pigs were divided into 3 groups with 18 pigs each. After a minimum of 1 week of acclimatization, the first group received a single oral infection of 10,000 embryonated *A. suum* eggs, the second group a trickle-infection of 1000 eggs/day for 10 days (10,000 eggs in total), and the last group served as an uninfected control group. The *A. suum* eggs were obtained from adult parasite specimens collected at an abattoir, allowed to embryonate at 25 °C for 2 months, and afterwards kept at 4 °C until oral inoculation of the pigs.

Successful infection was verified by anti-*Ascaris* antibody detection in serum samples taken weekly from each pig [human anti-*Ascaris lumbricoides* immunoglobulin G (IgG) enzyme-linked immunosorbent assay (ELISA), Abcam (Netherlands) B.V., Amsterdam, Netherlands].

**Fig. 1:**
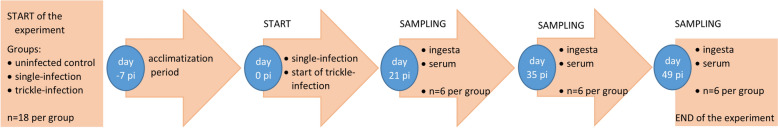
Experimental design and sampling strategy to investigate metabolomic changes during *Ascaris suum* infection

Six pigs each per group were slaughtered on days 21, 35, and 49 pi, respectively. Blood samples were taken during slaughter and centrifuged at 2000× *g* for 10 min at room temperature to obtain serum, which was stored at −80 °C until metabolomics analyses. Ingesta samples were taken with sterile tools from the proximal jejunum (approximately 3 m distal of the pylorus), medial jejunum (approximately 6–8 m distal of the pylorus), ileum, cecum, and colon ascendens (second coil); frozen immediately in liquid nitrogen; and stored at −80 °C for metabolomics analyses. The experimental setup and sampling scheme is visualized in Fig. 1.

### ^1^H NMR-based metabolomic assays

#### Sample preparation of ingesta and serum

Homorganic ingesta samples [100 mg; jejunum (proximal and medial), ileum, cecum, colon] were thawed for 30 min at 20 °C and moved to a polystyrene tray filled with ice. After addition of 800 µL MilliQ water and vortexing two times for 10 s at 4 m/s by using a bead mill homogenizer (Bead Ruptor Elite, OMNI International, NW Kennesaw, USA), samples were centrifuged at 14,000×*g* (Hettich centrifuge, Mikro 200R, Hettich GmbH, Tuttlingen, Germany) for 5 min at 4 °C. A volume of 350 µL of filtrate was transferred to a standard 5 mm glass NMR tube (Wilmad, LabGlass, USA), and 150 µL D_2_O (deuterium oxide; 99.9%, Cambridge Isotope Laboratories, Andover, MA), as well as 50 µL phosphate buffer (pH 7.4) containing 10 mM DSS [3-(trimethylsilyl)−1-propanesulfonic acid-d_6_ sodium salt, Sigma-Aldrich, St. Louis, MO] as internal standard for quantification and chemical shift reference, were added.

Serum samples (300 µL) were defrosted at 20 °C for 30 min, moved to a polystyrene tray with ice, and vortexed. Nanosep centrifugal filters with 3 kDa cutoff (Pall Life Science, Port Washington, NY, USA) were used to filter the samples after glycerol was removed from the filter membrane by washing 10 times with 0.5 mL MilliQ water (1500*g*, 36 °C, 15 min). Serum samples were filtered by centrifugation at 12,000×*g* for 1 h 30 min at 4 °C. To 200 µL serum filtrate, 50 µL D_2_O and 300 µL phosphate buffer (pH 7.4) containing 10 mM DSS were added and transferred to NMR tubes.

#### ^*1*^*H NMR spectroscopy and data processing*

All ingesta and serum samples were analyzed with a 400 MHz NMR spectrometer (Bruker BioSpin GmbH, Rheinstetten, Germany) operating at a proton NMR frequency of 400.31 MHz for ^1^H and equipped with a 5 mm TXI probe. Tuning and shimming was performed for each sample.

The 1D NOESY pulse experiment with presaturation of the spectral region containing the water peak (noesygppr1d) was performed at 300 K with 32 scans and 65,536 data points over a spectral width of 8223.69 Hz. Acquisition time was 3.98 s and an interscan relaxation delay of 6.5 s was used. ^1^H-^1^H correlation spectroscopy (COSY), ^1^H-^1^H total correlation spectroscopy (TOCSY), and ^1^H-^13^C heteronuclear single quantum correlation (HSQC) were obtained on one representative colon sample (trickle-infection, day 35 pi) for metabolite identification purposes. All data were processed using Bruker Topspin 4.0.7 software (Bruker BioSpin GmbH, Rheinstetten, Germany) and Fourier-transformed after multiplication by line broadening of 0.3 Hz. After the NMR spectra were manually phase and baseline corrected, each NMR spectrum was further analyzed using MATLAB (version R2018, MathWorks Inc., Natic, MA, USA). In MATLAB, data were referenced to standard peak DSS at 0.00 ppm and normalized to the area of DSS. The NMR spectra were binned into 0.01 ppm integral regions (buckets) between 9.0 ppm and 0.5 ppm (the area between 5.0 and 4.7 ppm containing the residual water signal was excluded). Afterward, the NMR signals were identified using ChenomX NMR Suite 8.4 library (ChenomX Inc., Edmonton, AB, Canada), the Human Metabolome Database (www.hmdb.ca) and previous literature (e.g., Wagner et al. [23] and Bankefors et al. [26]) and confirmed with 2D-NMR in case of multiplicity. Additionally, for quantification (profiling approach), 42 metabolites for ingesta (ileum, cecum, colon) and 66 for serum were identified by overlapping with standard spectra, and their concentrations (µmol/mg for ileum, cecum, and colon and µmol/ml for serum samples) were calculated using ChenomX NMR Suite version 8.4 profiler after accounting for overlapping signals.

#### Multivariate and univariate statistical analyses

Multivariate data analyses for the preprocessed ^1^H NMR spectral data (buckets, proximal and medial jejunum, ileum, cecum, colon, and serum) and for the absolute concentrations of ileum, cecum, colon, and serum metabolites (profiling approach) was performed using SIMCA-P + software (version 18.0, Umetrics AB, Umeå, Sweden). Both spectral data analysis (buckets) and absolute concentrations (profiling) were used complementarily to maximize insight. The untargeted spectral approach captures the full metabolic profile, including unknown or overlapping metabolites, while the targeted absolute quantification provides precise, physiologically relevant measurements to validate key findings and enable robust statistical testing, balancing comprehensive discovery with biological relevance. All variables were centered and “pareto”-scaled (spectral data) or “unit variance”-scaled (absolute concentrations data). Principal component analysis (PCA) was used to gain an overview of the data and identify outliers using PCA-Hotelling T^2^ Ellipse [95% confidence interval (CI)] and DModX (95% CI). The multivariate data were checked by normal probability plot of the PCA model for normal distribution. Orthogonal partial and partial least squares projection to latent structures-discriminant analysis (OPLS-DA and PLS-DA, respectively), which are supervised techniques, were performed for classification of the different samples at each timepoint according to experimental group. OPLS-DA loading plot and variable influences in projection (VIP) plot for spectral regions with VIP > 1 and with jack-knife-based CI that did not include unity were considered to be discriminative between the different groups. Cross validation (CV) analysis of variance (ANOVA) (*P* ≤ 0.05) for significance testing of OPLS-DA model was used [27]. Note that the term “CV-ANOVA” used in the context of OPLS-DA model validation refers to cross-validated analysis of variance for multivariate model assessment (as implemented in SIMCA-P +) and is distinct from univariate ANOVA procedures.

Additionally, the absolute concentrations of metabolites were tested for differences between the three groups at each day pi using the statistical software JMP (17.0.0, SAS Institute Inc., Cary, NC, USA). For univariate statistical testing of absolute metabolite concentrations, data normality was assessed using the Shapiro–Wilk test. Due to nonnormal distribution in multiple metabolites, parametric ANOVA was not applied. Instead, the nonparametric Kruskal–Wallis test was used to compare the three treatments at each day pi separately. When this test showed a significant effect (*P* ≤ 0.05), post hoc pairwise comparisons between the three groups (control versus single-infected, control versus trickle-infected, and single-infected versus trickle-infected) at each day pi were conducted by multiple Mann–Whitney tests.

All data presented are mean ± standard deviation (SD) and differences were considered statistically significant when *P* ≤ 0.05.

## Results

### Infection trial

No mortality occurred throughout the study period. ELISA results showed that most animals seroconverted between day 7 and 21 pi and a minority after day 21 pi [13], but 5 (two single- and three trickle-infected) of the 36 *A. suum*-infected animals had to be excluded from the study, since they did not seroconvert until slaughter and had no visible worms in the intestines [17]. All control animals remained seronegative during the study period.

There were no significant differences in daily weight gain between the experimental groups, except in the last cohort slaughtered on day 49 pi, with significantly higher daily weight gain in the trickle-infected group compared with the single-infected and the uninfected control group [17].

### ^1^H NMR-based metabolomic analysis

**Fig. 2:**
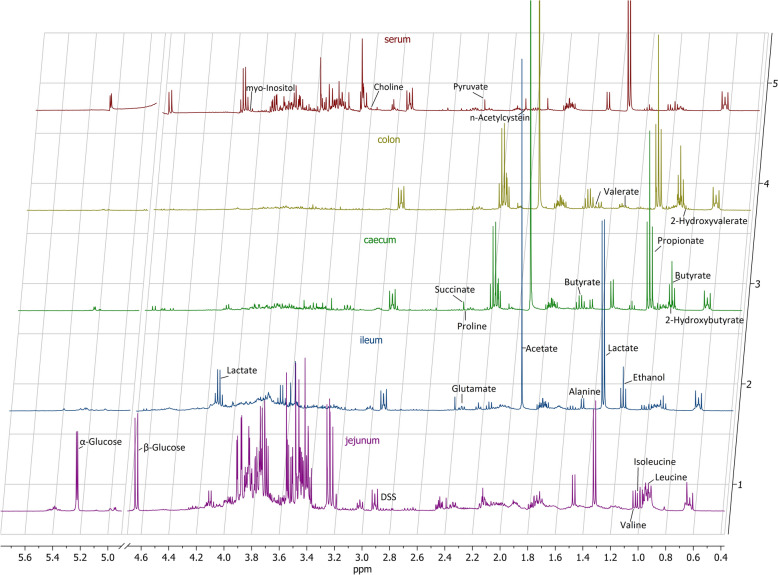
Representative 400 MHz ^1^H NMR spectra of jejunum, ileum, cecum, colon, and serum extracts from an *Ascaris suum* trickle-infected pig slaughtered on day 35 pi with assignment of important metabolites

Representative ^1^H NMR spectra of jejunum, ileum, cecum, colon, and serum extracts from a trickle-infected pig slaughtered on day 35 pi are shown in Fig. 2. To check for variation of metabolic profiles between *A. suum*-infected and uninfected control samples as well as between different parts of the ingesta and serum, the data obtained from NMR spectra were analyzed by PCA (PC1 versus PC2). The dataset comprised 241 ingesta samples and 51 serum samples (Table 1). Ingesta samples were collected from different anatomical sites: jejunum (96 samples from proximal and medial regions), ileum (47 samples), cecum (49 samples), and colon (49 samples). In detail, six pigs each from the uninfected control group contributed samples from each compartment at each day pi, with the exception of only five available ileum samples for day 35 pi. From the single infected group, four pigs each contributed proximal jejunum, ileum, cecum, and colon samples and five pigs medial jejunum samples at day 21 pi, while five samples were obtained from the ileum at day 35 pi and six samples each from jejunum, ileum, cecum, and colon at the remaining timepoints. From trickle-infected pigs, five samples per timepoint and anatomical location were included, except for only three and four available samples from proximal jejunum at day 21 pi and day 49 pi, respectively. Serum samples from six pigs per timepoint and group (single-infected, trickle-infected, and uninfected control) were included, except for day 49 pi with only five samples from each group. All model parameters (number of components, *R*^2^X, Q^2^, *R*^2^Y, CV-ANOVA) of PCA, OPLS-DA, or PLS-DA models are presented in Additional file 1.

**Table 1:** Overview of final sample numbers per group, timepoint, and anatomical compartment for ingesta and serum samples used for metabolomics analyses

Sample type/anatomical site	Group	Samples per timepoint		
		Day 21 pi	Day 35 pi	Day 49 pi
Ingesta				
Proximal jejunum (total *N* = 46)	Uninfected control	6	6	6
	Single-infected	4	6	6
	Trickle-infected	3	5	4
Medial jejunum (total *N* = 50)	Uninfected control	6	6	6
	Single-infected	5	6	6
	Trickle-infected	5	5	5
Ileum (total *N* = 47)	Uninfected control	6	5	6
	Single-infected	4	5	6
	Trickle-infected	5	5	5
Cecum (total *N* = 49)	Uninfected control	6	6	6
	Single-infected	4	6	6
	Trickle-infected	5	5	5
Colon (total *N* = 49)	Uninfected control	6	6	6
	Single-infected	4	6	6
	Trickle-infected	5	5	5
Serum [total *N* = 51 (49)*]	Uninfected control	6	6 (5)*	5
	Single-infected	6	6 (5)*	5
	Trickle-infected	6	6	5

^*^Outliers found in PCA plot, removed from further analyses

#### Metabolic changes in the ingesta

**Fig. 3:**
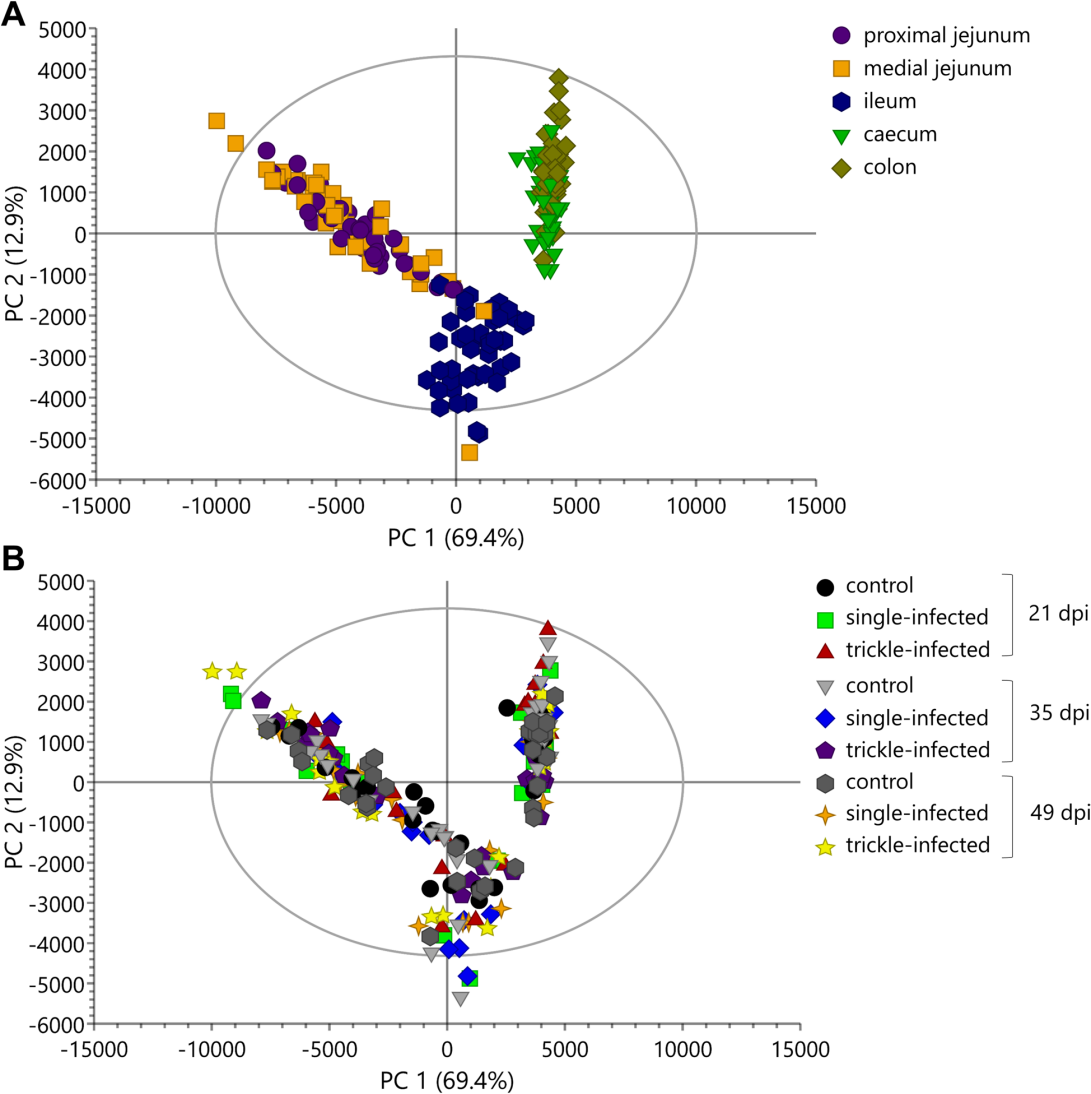
Principal component analysis (PCA) score scatter plots from ^1^H NMR spectrum profiles of *Ascaris suum* infected and uninfected control pigs, colored according to the intestinal compartment (**A**) and the different experimental groups (**B**)

In the PCA model, the first (horizontal) and second (vertical) components explained 69.4% and 12.9% of variation, respectively, and the total amount of X variation was 99.0% using 24 principal components (Fig. 3A). A clear separation between the different intestinal compartments, but not between the experimental groups nor between the different timepoints (Fig. 3B), was observed in the PCA score scatter plot. An OPLS-DA and PLS-DA model, which are supervised models to confirm the results obtained by PCA, resulted in no significant difference between the experimental groups in the overall dataset. Therefore, the data were further analyzed individually for each intestinal compartment.

##### Ingesta: Jejunum

The PCA model of all jejunum (proximal and medial) samples was described by 17 PCs, whereof the first and second component explained 55.0% and 20.1% of variation, respectively, and showed no clear separation between the *A. suum*-infected groups and the uninfected control group (Additional file 2). Similar results were observed in the OPLS-DA model. Further investigations by considering each day pi separately (e.g., single-/trickle-infected versus uninfected control on day 21 pi, day 35 pi, and day 49 pi) also did not reveal any significant differences in the PCA, OPLS-DA, and PLS-DA models. Therefore, no further data analyses were performed for jejunum samples.

##### Ingesta: Ileum

**Fig. 4:**
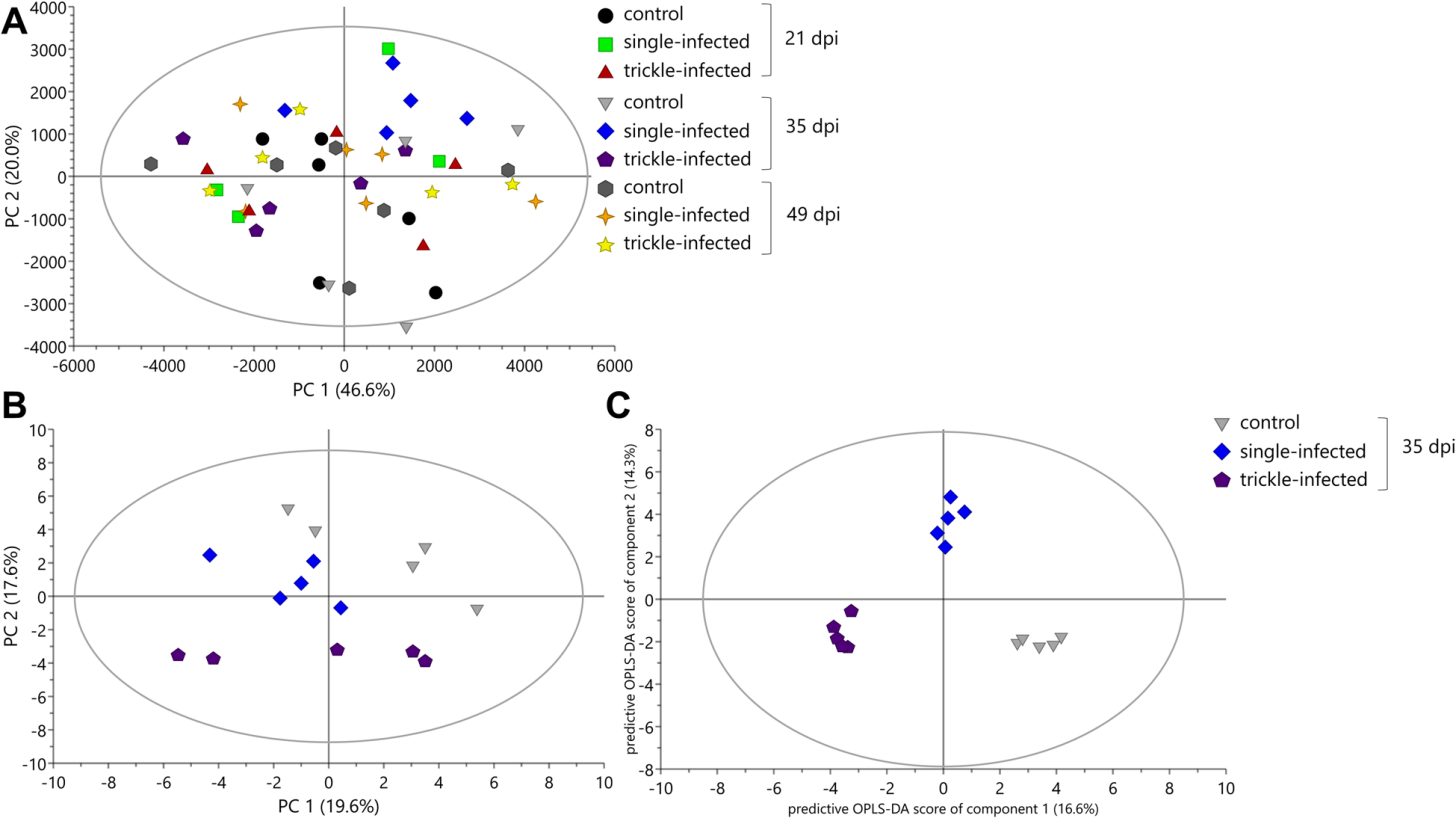
Principal component analysis (PCA; **A**, **B**) and orthogonal partial least squares projection to latent structures-discriminant analysis (OPLS-DA; **C**) score scatter plots from ^1^H NMR spectrum profiles of ileum samples from *Ascaris suum*-infected and uninfected control pigs

The PCA model for the ileum samples was explained by 10 PCs, whereof PC 1 explained 46.6% and PC 2 20.0% of variation (Fig. 4A). It was not possible to generate OPLS-DA and PLS-DA models due to a lack of clear group separation and insufficient predictive power for supervised classification methods, indicating that infection effects on these samples may have been subtle or variable. Further investigations by comparisons of the three groups for each day pi revealed no significant differences on day 21 and 49 pi, but both *A. suum*-infected groups showed significant differences compared with the uninfected control pigs on day 35 pi. To identify changes between the groups, the absolute concentrations of 42 metabolites were quantified through a profiling approach from ^1^H NMR spectra. The PCA model for all three groups on day 35 pi resulted in four components, with the first and second components explaining 19.6% and 17.6% of variation, respectively, and the total amount of X variation was 62.7% (Fig. 4B). A separation between the uninfected control group (Fig. 4B top) and single-infected (below, left side) as well as trickle-infected group (bottom) was observed and confirmed as statistically significant via OPLS-DA model (model parameters: *R*^2^X = 56.6%, *Q*^2^ = 75.1%, *R*^2^*Y* = 96.4%, 2 + 2 + 0 components, CV-ANOVA = 0.017) (Fig. 4C).

**Fig. 5:**
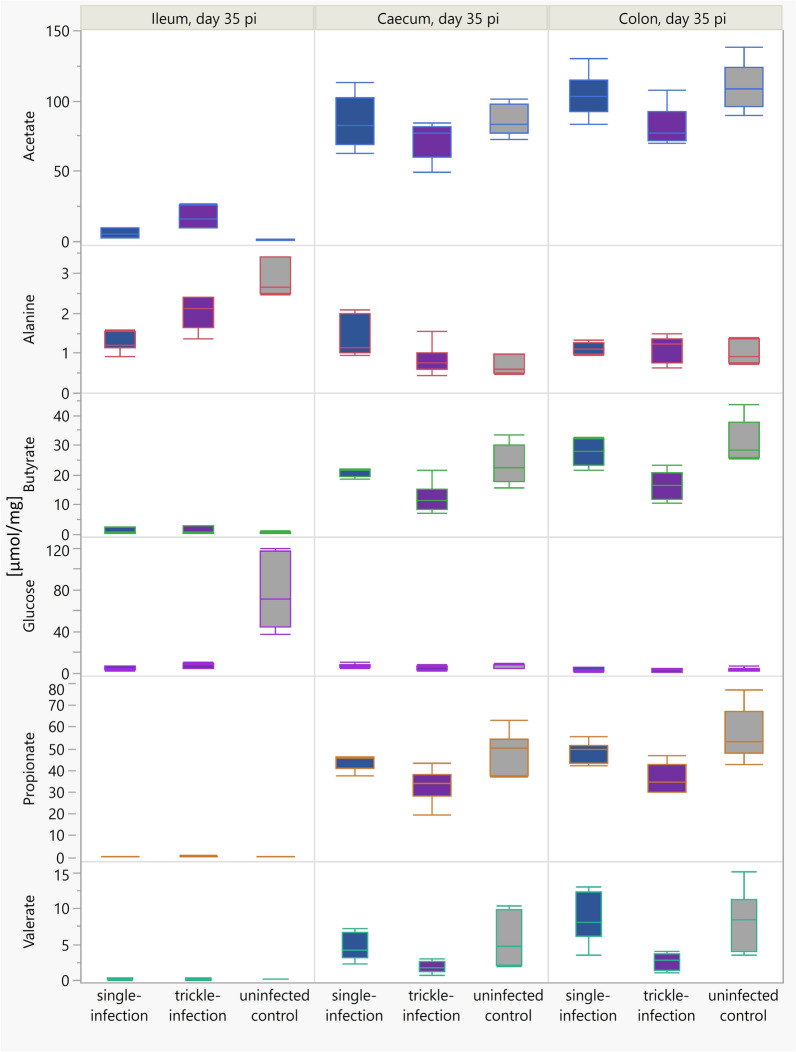
Boxplots of the most important metabolites in the ileum, cecum, and colon of *Ascaris suum*-infected pigs versus an uninfected control group on day 35 pi

To identify the metabolites driving the group separation, the VIP plot and loading column plot of OPLS-DA model were examined, and univariate comparisons of metabolite concentrations were subsequently performed (Kruskal–Wallis/Mann–Whitney tests). Higher concentrations of acetate and lactate were observed in the single-infected group on day 35 pi compared with the uninfected control group, but lower levels of glucose and amino acids (e.g., alanine, isoleucine, leucine, propionate, threonine, valine), while the trickle-infected group also showed higher levels of acetate, and lower levels of glucose and amino acids (e.g., alanine, glutamate, isoleucine, leucine, threonine, and valine) compared with the uninfected control group (Table 2 and Fig. 5).

**Table 2:** Statistically significant ingesta metabolite changes in *Ascaris suum* single- or trickle-infected pigs

Compartment	Single-infection versus uninfected control			Trickle-infection versus uninfected control		
	Day 21 pi (*P*-value)	Day 35 pi (*P*-value)	Day 49 pi (*P*-value)	Day 21 pi (*P*-value)	Day 35 pi (*P*-value)	Day 49 pi (*P*-value)
Jejunum	n.s.	n.s.	n.s.	n.s.	n.s.	n.s.
Ileum	n.s.	Acetate (0.005) ↑ Lactate (0.005) ↑ Alanine (0.005) ↓ Glucose (0.005) ↓ Isoleucine (0.005) ↓ Leucine (0.005) ↓ Propionate (0.013) ↓ Threonine (0.005) ↓ Valine (0.005) ↓	n.s.	n.s.	Acetate (0.005) ↑ Alanine (0.005) ↓ Glucose (0.005) ↓ Glutamate (0.005) ↓ Isoleucine (0.005) ↓ Leucine (0.020) ↓ Threonine (0.005) ↓ Valine (0.005) ↓	n.s.
Cecum	n.s.	Alanine (0.030) ↑ Aspartate (0.037) ↑ Glutamate (0.020) ↑ Propionate (0.014) ↓ Valine (0.045) ↑	n.s.	2-hydroxybutyrate (0.012) ↑ Pyruvate (0.022) ↓	Butyrate (0.008) ↓ 3-Hydroxyisobutyrate (0.008) ↓ 2-hydroxyvalerate (0.014) ↑ Proline (0.023) ↓ Propionate (0.020) ↓ Valerate (0.036) ↓	n.s.
Colon	Pyruvate (0.037) ↑	n.s.	2-hydroxybutyrate (0.013) ↑ 4-hydroxybutyrate (0.006) ↑ thymine (0.030) ↑	Acetate (0.014) ↑ Butyrate (0.022) ↑ choline (0.022) ↑ Glutamate (0.022) ↑ 2-hydroxybutyrate (0.022) ↑ 2-hydroxyvalerate (0.036) ↑ hypoxanthine (0.035) ↑ n-acetylcysteine (0.023) ↑ Proline (0.012) ↑ Pyruvate (0.020) ↑ Succinate (0.012) ↑ Tyrosine (0.011) ↑ Valerate (0.022) ↑	Acetate (0.014) ↓ Butyrate (0.008) ↓ 3-hydroxyisobutyrate (0.008) ↓ n-acetylcysteine (0.014) ↓ Phenylacetate (0.023) ↓ Propionate (0.018) ↓ Valerate (0.014) ↓	Choline (0.008) ↑ 2-hydroxybutyrate (0.008) ↑ 4-hydroxybutyrate (0.022) ↑ 2-hydroxyvalerate (0.012) ↑ n-acetylcysteine (0.008) ↑ Proline (0.014) ↑

Arrows indicate direction of change relative to the uninfected control group (↑ = increased, ↓ = decreased). Statistical analysis was performed using Kruskal–Wallis/Mann–Whitney test. Numbers in brackets are *P*-values, with *P* ≤ 0.05 considered significant

n.s., no statistically significant differences

##### Ingesta: Cecum

**Fig. 6:**
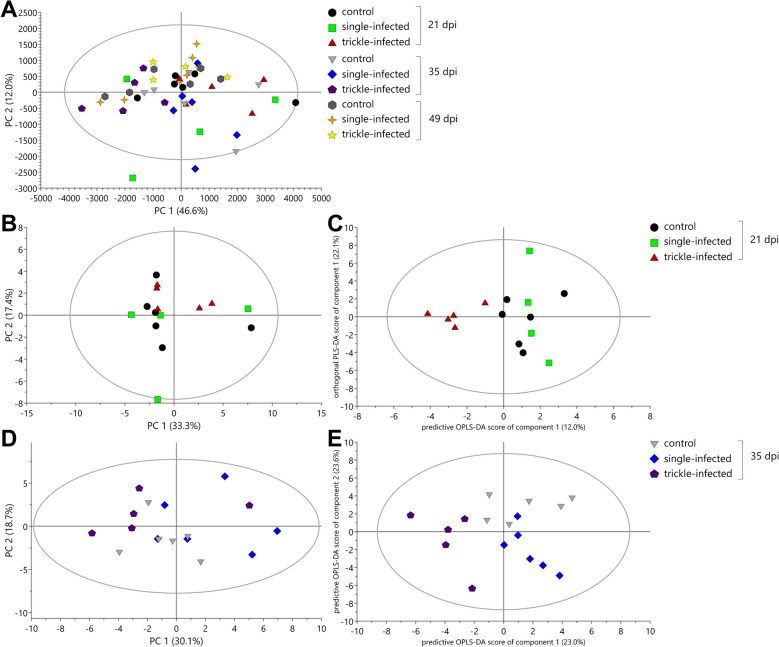
Principal component analysis (PCA; **A**, **B**, **D**) and orthogonal partial least squares projection to latent structures-discriminant analysis (OPLS-DA; **C**, **E**) score scatter plots from ^1^H NMR spectrum profiles of cecum samples from *Ascaris suum*-infected and uninfected control pigs

The PCA model for the cecum samples was described by eight PCs, whereof the first and second component explained 46.6% and 12.0% of variation, respectively (Fig. 6A). No PLS-DA, but an OPLS-DA model with one predictive and zero orthogonal components (1 + 0 + 0), were generated. As for the ileum samples, a comparison of the groups at the different timepoints was conducted. No differences were observed on day 49 pi. Visual inspection of the PCA score plot suggested a potential separation between the trickle-infected and the uninfected control groups at days 21 and 35 pi, therefore the profiling approach was used to identify changes between the groups. The fitted PCA model for day 21 pi was explained by two PCs, whereof the first explained 33.3% and the second explained 17.4% of variation (Fig. 6B). The score scatter plot of the OPLS-DA model using one predictive and one orthogonal component achieved separation between the trickle-infected group and the uninfected control group (Fig. 6C). The loading plot and VIP plot were used to identify the metabolites driving the differences at day 21 pi. The statistical analysis using Mann–Whitney test revealed lower concentrations of pyruvate and higher concentrations of 2-hydroxybutyrate in the trickle-infected group compared with the uninfected control group (Table 2).

In the PCA model for cecum samples from day 35 pi, the first and second components explained 30.1% and 18.7% of variation, respectively, and the total amount of X variation was 66.0% using three principal components (Fig. 6D). The investigation with the OPLS-DA model showed a clear separation between the three experimental groups along the horizontal and vertical axis, generating two predictive and zero orthogonal components (Fig. 6E). The statistical analyses via Mann–Whitney test revealed five metabolites with significantly different concentrations in the single-infected group versus uninfected control group. Alanine, aspartate, glutamate, and valine were increased compared with the uninfected controls, whereas propionate was significantly decreased. With regard to the trickle-infected group, six metabolites were identified as significantly different, with higher concentrations of 2-hydroxyvalerate, and lower concentrations of butyrate, 3-hydroxyisobutyrate, proline, propionate, and valerate compared with the uninfected control group (Table 2 and Fig. 5).

##### Ingesta: Colon

**Fig. 7:**
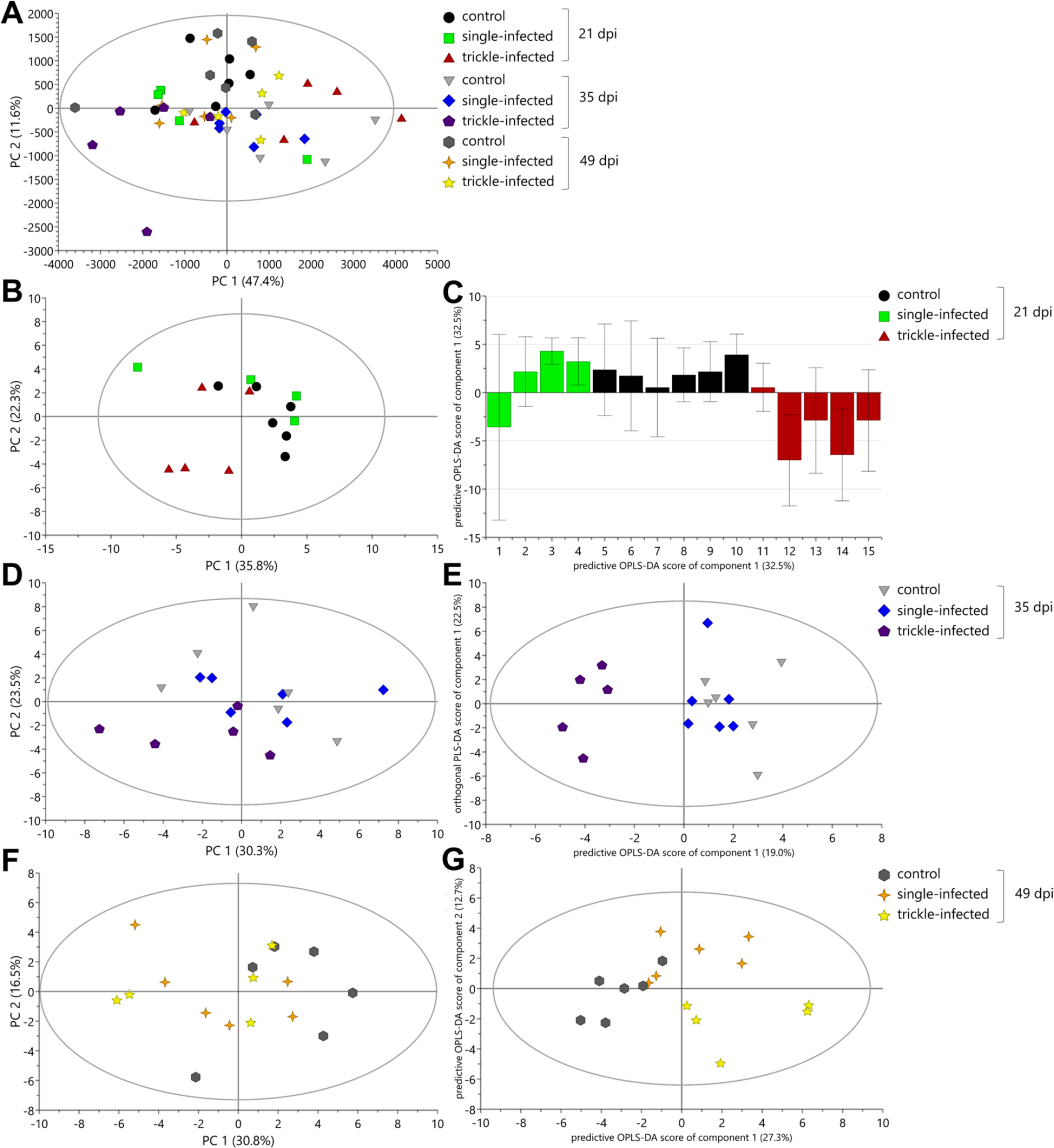
Principal component analysis (PCA; **A**, **B**, **D**, **F**) and orthogonal partial least squares projection to latent structures-discriminant analysis (OPLS-DA; **C**, **E**, **G**) score scatter plots from ^1^H NMR spectrum profiles of colon samples from *Ascaris suum*-infected and uninfected control pigs

The PCA model of all colon samples was described by 12 PCs. The first and second principal components explained 47.4% and 11.6%, respectively, of spectral variation in the PCA model (Fig. 7A). One PLS-DA and an OPLS-DA model with two predictive and zero orthogonal components (2 + 0 + 0) were generated. Both models showed differences between the three groups, and further investigations for each day pi were conducted using the profiling approach.

In the PCA model for day 21 pi, the first and second components explained 35.8% and 22.3%, respectively, of spectral variation using three PCs (Fig. 7B). Further investigations via OPLS-DA model showed one predictive component (Fig. 7C) clearly separating the trickle-infected group from the uninfected control group. The statistical analyses revealed a higher concentration of pyruvate in the single-infected compared with the uninfected control group, whereas a total of 13 metabolites was increased in the trickle-infected group on day 21 pi, namely acetate, butyrate, choline, glutamate, 2-hydroxybutyrate, 2-hydroxyvalerate, hypoxanthine, n-acetylcysteine, proline, pyruvate, succinate, tyrosine, and valerate (Table 2).

In the PCA score scatter plot for day 35 pi, the first component described 30.3% of variation, while the second component explained 23.5% of spectral variation (Fig. 7D). The data showed a tendency toward a separation of the trickle-infected group (bottom) and the uninfected control group (top) in the vertical axis. Further analysis with the OPLS-DA model resulted in one predictive and one orthogonal component (Fig. 7E). The model revealed discriminating metabolites that tended to differ between the trickle-infected and the uninfected control group. The statistical analyses confirmed seven significantly decreased metabolites in the trickle-infected group compared with the uninfected control group: acetate, butyrate, 3-hydroxyisobutyrate, n-acetylcysteine, phenylacetate, propionate, and valerate (Table 2 and Fig. 5). In contrast, no significant differences were observed between the single-infected versus uninfected control group in the colon on day 35 pi.

The fitted PCA model for day 49 pi was explained by three PCs, whereof PC 1 explained 30.8% and PC 2 explained 16.5% of variation (Fig. 7F). The score scatter plot of the OPLS-DA model using two predictive and zero orthogonal components (Fig. 7G) achieved a separation between the uninfected control group (Fig. 7G, left side), the single-infected (right side, top), and the trickle-infected group (right side, bottom). Higher concentrations of 2-hydroxybutyrate, 4-hydroxybutyrate, and thymine were observed in the single-infected group compared with the uninfected control group. In the trickle-infected group, 2-hydroxybutyrate and 4-hydroxybutyrate were also increased, in addition to choline, 2-hydroxyvalerate, n-acetylcysteine, and proline (Table 2).

#### Metabolic changes in serum

**Fig. 8:**
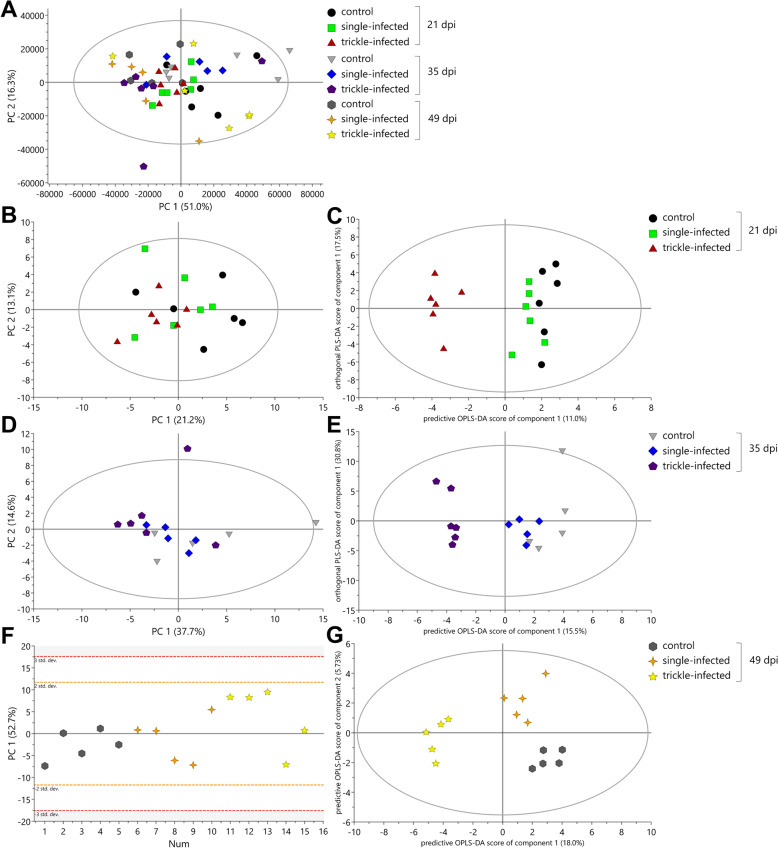
Principal component analysis (PCA; **A**, **B**, **D**, **F**) and orthogonal partial least squares projection to latent structures-discriminant analysis (OPLS-DA; **C**, **E**, **G**) score scatter plots from ^1^H NMR spectrum profiles of serum samples from *Ascaris suum*-infected and uninfected control pigs

Two outliers exceeding both the Hotelling’s T^2^ statistic and DModX threshold were found in the serum samples of day 35 pi (from one single-infected and one uninfected control pig) and were excluded from further analysis, as they most likely represented technical artifacts. The analysis of the remaining 49 serum samples resulted in a 9-component model that explained 93.3% of the total amount of X variation. The first PC accounted for 51.0% of the total variance and the second component for 16.3% (Fig. 8A). The data analyses showed a tendency of separation between the trickle-infected and the uninfected control group in regard to day pi. Further analyses via PLS-DA and OPLS-DA resulted in none or two predictive and zero orthogonal (2 + 0 + 0) components, respectively. On the basis of the observed differences between the groups, further data analyses were conducted between the three groups for each day pi using a profiling approach.

In the PCA score scatter plot for day 21 pi, the first and second PCs explained 21.2% and 13.1%, respectively, of spectral variation (Fig. 8B). The OPLS-DA score scatter plot model using one predictive and one orthogonal component separated the trickle-infected group (left side) clearly from the uninfected control group (right side) (Fig. 8C). A total of 11 metabolites were identified as significantly different between the trickle-infected and uninfected control group, with higher concentrations of butyrate and lower concentrations of creatinine, dimethylsulfone, glucose, glutamine, glycine, myo-inositol, n-acetylcysteine, ornithine, propylene glycol, and pyroglutamate in the trickle-infected group (Table 3). No significant differences were observed between the single-infected and the uninfected control group on day 21 pi.

**Table 3:** Statistically significant serum metabolite changes in *Ascaris suum* single- or trickle-infected pigs

Single-infection versus uninfected control			Trickle-infection versus uninfected control		
Day 21 pi	Day 35 pi	Day 49 pi	Day 21 pi (*P*-value)	Day 35 pi (*P*-value)	Day 49 pi (*P*-value)
n.s.	n.s.	n.s.	Butyrate (0.041) ↑ Creatinine (0.008) ↓ Dimethylsulfone (0.010) ↓ Glucose (0.012) ↓ Glutamine (0.023) ↓ Glycine (0.030) ↓ myo-inositol (0.008) ↓ n-acetylcysteine (0.010) ↓ Ornithine (0.014) ↓ Propylene glycol (0.020) ↓ Pyroglutamate (0.005) ↓	Alanine (0.012) ↓ β-alanine (0.014) ↓ Citrate (0.014) ↓ Glucose (0.012) ↓ Glutamine (0.02) ↓ Lactate (0.012) ↓ Methionine (0.012) ↓ myo-inositol (0.020) ↓ n-acetylcysteine (0.012) ↓ Pyroglutamate (0.008) ↓ Pyruvate (0.037) ↓ Trimethylamine n-oxide (0.007) ↓	Acetate (0.012) ↑ Asparagine (0.020) ↑ Butyrate (0.016) ↑ Glutamine (0.037) ↑ 3-hydroxybutyrate (0.019) ↑ Propylene glycol (0.020) ↑ Pyruvate (0.020) ↑

Arrows indicate direction of change relative to the uninfected control group (↑ = increased, ↓ = decreased). Statistical analysis was performed using Kruskal–Wallis/Mann–Whitney test. Numbers in brackets are *P*-values, with *P* ≤ 0.05 considered significant

n.s., no statistically significant differences

In the PCA score scatter plot for day 35 pi, the first component described 37.7%, while the second component explained 14.6% of spectral variation (Fig. 8D), after removing the abovementioned outliers. The score scatter plot of the OPLS-DA model using one predictive and two orthogonal components (Fig. 8E) achieved a separation between the trickle-infected group (Fig. 8E, left side) and the uninfected control group (right side). A total of 12 metabolites showed significantly decreased concentrations in the trickle-infected group (Table 3). As on day 21 pi, glucose, glutamine, myo-inositol, n-acetylcysteine, and pyroglutamate were decreased, among others. No significant differences were observed between the single-infected and the uninfected control group on day 35 pi.

The PCA score scatter plot for day 49 pi resulted in one component that described 52.7% of variation (Fig. 8F). Further investigation with OPLS-DA model resulted in two predictive components, explaining 18.0% and 5.73% of variation, respectively, and two orthogonal components (Fig. 8G). The OPLS-DA model showed a clear separation of the three experimental groups. The statistical analyses via Mann–Whitney test revealed seven significantly increased metabolites in the trickle-infected group compared with the uninfected control group, among others, acetate, asparagine, butyrate, and 3-hydroxybutyrate (Table 3). In contrast, no significant differences were observed for the single-infected group on day 49 pi.

## Discussion

The use of NMR-based metabolomics in the present study demonstrated changes in the metabolic profile of the ileum, cecum, and colon contents as well as serum samples of both *A. suum* single- and trickle-infected pigs during the early infection phase. Such metabolic changes may be due to the worms’ own metabolic activity, infection-induced alterations to the intestinal microbiota, or modulated nutrient absorption by the host.

The metabolomics analyses revealed generally more pronounced changes in the trickle-infected compared with the single-infected pigs. This is in accordance with previous findings regarding functional measurements of intestinal nutrient transport in the same experimental animals, which also showed stronger alterations in the trickle- than single-infected group [13]. This difference may be attributed to higher worm burdens in the trickle-infected animals, as a single-infection with high egg numbers can trigger a strong self-cure reaction in the host, resulting in the expulsion of most larvae [8, 28]. Importantly, trickle-infections are a better reflection of how pigs are naturally exposed to roundworms, making them more biologically relevant. Unfortunately, it was not possible to assess individual infection intensity in this pilot study, as the intestines were subjected to electrogenic nutrient transport measurements immediately after slaughter, precluding intestinal washing to recover all parasites [13]. Therefore, further studies are necessary to investigate the quantitative relationship between worm burden and metabolic changes.

### Compartment-specific metabolic alterations

Despite the jejunum being the primary site of worm establishment, we observed no metabolic changes in this compartment throughout the study. To the authors’ knowledge, no previous studies on metabolic changes in the small intestine during *A. suum* infection have been conducted. When examining functional nutrient transport of the hosts’ small intestinal mucosa, only limited changes were observed in the *Ascaris*-infected versus the control group [13]. However, metabolic changes due to the worms’ own metabolic activity or microbial alterations were expected. An investigation into the jejunal microbiota of the same animals was unfortunately inconclusive due to low sequencing success of samples from this compartment [17]. Therefore, this unexpected finding needs further investigation into the mechanism of local metabolic adaptation at the parasite colonization site.

In the other intestinal compartments, metabolic changes that showed a clear temporal pattern were observed, with pronounced alterations at day 35 pi that were largely absent at day 49 pi. This probably reflects the different phases of *A. suum* infection, with day 35 pi characterized by active larval development and rapid worm growth, while worms have matured by day 49 pi and the host has adapted metabolically.

In the ileum, we detected significantly higher concentrations of lactate (in single-infected pigs) and acetate (single- and trickle-infected pigs) on day 35 pi. The observed increase in lactate is consistent with previous findings showing that the lactate producing *Lactobacillus* spp. are elevated during many helminth infections. This was observed in pigs infected with *A. suum*, with an expansion of the genus *Lactobacillus* in the jejunum and colon on day 14 pi [29]. The increase might be related to decreased lactase activity during *A. suum* infection [30], also reflected by lactose intolerance in children infected with *A. lumbricoides* [31], higher levels of lactose in intestinal contents of infected pigs, or increased mucus secretion during helminth infection that promotes the growth of lactobacilli [29]. According to Iraporda et al. [32], lactate may reduce gut proinflammatory responses, which may be beneficial for both the host and the parasite. The increased acetate levels are in line with the findings of Tielens et al. [33], who identified acetate as a primary end product of energy metabolism in parasitic helminths, such as *A. suum*. In vivo evidence of how parasite metabolism affects the host metabolome during *A. suum* infection can be found in the observed temporal patterns of acetate fluctuations in the present study, which most likely reflect parasite development stages, with molting of L4 to preadult stages occurring approximately days 21–29 pi, and host–parasite metabolic interactions.

In addition to the observed alterations in acetate and lactate, the analysis of the ileal metabolome showed interesting changes in the amino acid profile of *A. suum*-infected pigs, indicating a more extensive metabolic effect of the parasite on host nutrient absorption and utilization. At day 35 pi, a significant decrease in several amino acids, including alanine, glutamate, isoleucine, leucine, threonine, and valine, was observed in the ileum of the infected animals. Leucine, isoleucine, and valine are branched-chain amino acids (BCAAs), which are important for energy production and protein synthesis. The observed decrease in ileal amino acid concentrations may indicate enhanced utilization of these nutrients, either by the host or by the parasites themselves. When conducting functional measurements, no significant changes in intestinal amino acid and peptide transport across the infected pigs’ ileal mucosa were noted on day 35 pi, whereas the transport was reduced on day 49 pi [13]. Thus, increased absorption by the host is an unlikely cause of the reduced amino acid concentrations in the ileum content, whereas it may be speculated that the growing worms’ demand for amino acids is the underlying driver. When cultivating adult *A. suum* in vitro, absorption of the mentioned amino acids was shown, with the exception of alanine, which was secreted into the culture medium, probably as a metabolic end product [14]. Of particular interest is the decrease in glutamate and BCAAs observed in the present study, which according to Zhang et al. [34], are essential for pig health and disease resistance. In addition, these nonessential amino acids might not be synthesized in sufficient amounts during health challenges [34]. Thus, *A. suum* infection could result in a conditionally essential need for some amino acids that are normally classified as nonessential, which could affect the immune system, protein synthesis, and general growth performance in infected animals.

Furthermore, increased absorption by the helminths may also account for the decreased glucose concentrations of infected pigs seen in the ileum on day 35 pi, similar to amino acids [14]. This ileal nutrient depletion represents the competitive nutrient acquisition between the parasite and host and may be a contributing factor to the observed serum metabolic changes, especially in trickle-infected animals.

In the cecum of single-infected pigs, we observed significantly higher concentrations of certain amino acids (alanine, aspartate, glutamate, and valine) on day 35 pi. Interestingly, this amino acid profile was different from the trickle-infected group, suggesting that different metabolic reactions depend on the infection protocol. The antimicrobial properties of *A. suum* products, as reported by Midha et al. [35], may account for this compartment-specific increase in amino acid concentrations in the cecum, which contrasts with the lower levels found in the ileum. Their study showed that a variety of antibacterial factors, such as glycosyl hydrolases, cecropins, and members of *A. suum* antibacterial factors (ASABFs), are present in *A. suum* excretory–secretory products. These compounds most likely produce a gradient of antimicrobial activity that decreases in the cecum and is highest in the adult worms’ primary habitat. The development of unique microbial communities in the cecum with improved capacity for protein fermentation and amino acid production may be encouraged by such differential antimicrobial pressure. The increase may also be the result of changes in the small intestine’s processes for digesting and absorbing proteins, which would raise the flow of amino acids into the large intestine.

By day 35 pi, butyrate, propionate, and valerate concentrations in the cecum of trickle-infected pigs were noticeably lower than in the control group, in line with the decline in SCFA-producing bacterial abundance in the cecum at this timepoint [17]. Interestingly, more pronounced microbial changes were found in this compartment than metabolic ones, whereas the opposite was found for the colon, which showed the most pronounced metabolic changes of all intestinal compartments analyzed, but only little microbial changes [17]. This suggests compartment-specific patterns in the response to infection, with the cecum showing predominantly microbial community restructuring and the colon showing predominantly metabolic alterations. Whether these compartment-specific changes represent host adaptive responses to mitigate infection impact, parasite-induced alterations that facilitate parasitism, or simply direct consequences of the infection, remains unclear and warrants further investigation.

On day 21 pi, we observed a significant increase of SCFAs, including acetate, butyrate, and valerate, as well as amino acids (glutamate, proline, tyrosine) in the colon of trickle-infected pigs compared with the uninfected control group. Our findings temporally extend the observations made by Zaiss et al. [18] at 8 weeks pi, demonstrating that SCFA elevation occurs already at day 21 pi. As reviewed by Midha et al. [19], such SCFA increases are common across various helminth infections and likely create an environment that favors parasite establishment through immunomodulatory effects.

Since succinate is an essential intermediate metabolite in bacterial fermentation pathways, its simultaneous elevation with several SCFAs at day 21 pi is especially noteworthy [36]. The simultaneous increase in pyruvate and glutamate, which are implicated in succinate-acetate pathways and the γ-aminobutyric acid (GABA) shunt pathway, respectively, suggests a major shift in colonic microbial metabolism during *A. suum* trickle-infection. The succinate–acetate pathway represents a key fermentation route where succinate serves as an intermediate in the conversion of complex carbohydrates to acetate, while the GABA shunt pathway involves the conversion of glutamate to GABA and subsequently to succinate, providing an alternative route for energy metabolism in certain bacterial species. These metabolic changes most likely reflect changes in the metabolic activity rather than major shifts in bacterial abundance, as our microbiome analysis showed limited compositional changes in the colon. Key bacterial taxa involved in these fermentation pathways include *Bacteroides* spp., *Prevotella* spp., and members of the Firmicutes phylum, which are known producers of SCFAs through succinate–propionate and succinate–butyrate pathways [37, 38]. According to the overall metabolic profile, an *A. suum* infection causes a coordinated reorganization of the fermentative capacity of the gut microbiota, potentially altering both protein digestion and absorption processes.

The observed increase in n-acetylcysteine (NAC) in the colon of trickle-infected pigs on days 21 and 49 pi is noteworthy. NAC is known for its antioxidant and antiinflammatory properties in the gastrointestinal tract, including the ability to reduce reactive oxygen species (ROS) production and modulate inflammatory responses [40]. The increase in NAC observed in our study may reflect a host response to infection-induced oxidative stress and inflammation in the colonic environment. However, the precise functional significance of elevated NAC levels during *A. suum* infection and its potential interaction with other metabolic changes, such as the observed alterations in SCFAs, requires further investigation. By day 35 pi, the colon contents of trickle-infected pigs showed significantly lower levels of a number of metabolites, including acetate, butyrate, propionate, valerate, NAC, 3-hydroxyisobutyrate, and phenylacetate. In contrast, by day 49 pi, increased levels of 2-hydroxybutyrate, 4-hydroxybutyrate, 2-hydroxyvalerate, choline, proline, and NAC were observed in the colon. Again, this temporal pattern indicates that intestinal metabolic changes occur in a dynamic pattern, in line with larval development and worm establishment, suggesting a partial recovery and possible adaptation in the host metabolic profile.

The significant elevation of choline at both day 21 and day 49 pi in the colon of trickle-infected pigs also represents a noteworthy finding. According to Xie et al. [40], choline is an essential nutrient affecting pig growth performance and carcass characteristics. Their study showed that short-term choline chloride supplementation resulted in a significant increase in carcass weight and final weight. Interestingly, our findings show a temporal coincidence between elevated choline levels at day 49 pi and the noticeably higher weight gain reported by Springer et al. [17] in trickle-infected pigs at the same timepoint. This suggests that the *A. suum*-induced increase in choline levels could be an adaptive mechanism that improves growth performance in spite of parasitic burden. The biphasic nature of choline elevation (at days 21 and 49, with an interim normalization at day 35) likely reflects the host–microbiome system’s dynamic adaptation during the course of infection, potentially impeding the negative effects of chronic worm infection on growth and nutrient utilization.

### Serum metabolic changes

In single-infected pigs, no significant changes were observed in serum samples throughout the study, further supporting the notion that the metabolic impact of *A. suum* is dose dependent and influenced by the infection pattern.

In the samples of trickle-infected pigs, we observed lower concentrations of glucose on days 21 and 35 pi, as well as lower levels of alanine on day 35 pi, among other metabolites, in comparison with the control group. The reduced concentrations of glucose and alanine support previous findings of intestinal nutrient transport restriction [13, 14]. Interestingly, we also observed a decrease in pyruvate at day 35 pi followed by its increase at day 49 pi, which further supports disturbances in central energy metabolism. Pyruvate plays a particularly important role because it connects key metabolic pathways such as glycolysis, the tricarboxylic acid (TCA) cycle, and amino acid metabolism. The observed simultaneous reduction in serum glucose, alanine, and pyruvate on day 35 pi in the trickle-infection group is consistent with the timing of rapid parasite growth and peak metabolic demand. At this timepoint, developing worms are actively growing and require substantial nutrient uptake, while the host immune response represents an additional metabolic burden on the host. This suggests a broad disruption of energy-generating pathways where the infection affects more than just glucose absorption. By day 49 pi, when parasites have reached maturity and growth rates have slowed, pyruvate levels increased, suggesting reduced metabolic pressure and potential host metabolic adaption to the chronic infection state. In response to infection, Koehler et al. [13] showed a significant upregulation of transcription factors STAT6 and Hif-1α, as well as glucose and peptide transporters. This molecular adaptation points to a compensatory mechanism to counteract metabolic disruptions brought on by parasite infection. The development of the protective Th2-dominated immune response, which is marked by elevated IgE production, eosinophilia, and mast cell-dependent hypersensitivity reactions in the small intestine, occurs at the same time as these metabolic alterations [12]. Interestingly, Dawson et al. [12] found an increase in Na-coupled glucose absorption, along with enhanced smooth muscle contractility and mucosal mast cell hyperplasia at 14 and 21 days after infection. Despite the upregulation of glucose absorption capacity reported by Dawson et al. [12], our observed decrease in serum glucose indicates that this compensatory mechanism is insufficient to meet the combined metabolic demands of the parasites and the activated immune system.

As the infection progressed, a clear pattern emerged in the temporal changes of serum metabolites. We observed a notable shift toward increased metabolite levels (e.g., 3-hydroxybutyrate, acetate, asparagine, butyrate, glutamine, propylene glycol, and pyruvate) on day 49 pi, possibly indicating metabolic recovery or adaptation, whereas days 21 and 35 pi were mainly marked by decreased metabolite concentrations, suggesting a catabolic state. Particularly significant is the simultaneous recovery of pyruvate and increased 3-hydroxybutyrate at day 49 pi, which points to a possible metabolic shift toward alternate energy pathways in reaction to the prior glucose deficiency. This metabolic adaptability might be a crucial component of the host’s resistance to long-term parasitic infection. The metabolic alterations observed in the colon, where SCFAs and other metabolites were increased at day 21 pi, decreased at day 35 pi, and partially restored by day 49 pi, are mirrored in this triphasic pattern. For example, we observed increased butyrate levels in serum at days 21 and 49 pi, mirroring the colonic SCFA dynamics, indicating effective absorption of this metabolite produced by microbes.

The observed NAC decrease in serum at days 21 and 35 pi is consistent with its decrease in the colon at day 35 pi, indicating compartment-specific roles for this antioxidant, but it contrasts with its elevation in the colon at days 21 and 49 p. According to Wang et al. [39], this differential pattern may be the result of upregulating local intestinal antioxidant mechanisms while concurrently using more systemic antioxidant reserves to fight infection-induced oxidative stress. The subsequent increase in serum metabolites at day 49 pi suggests a possible resolution phase, during which local and systemic metabolic processes start to return to normal or adjust to the parasite’s ongoing presence.

Myo-inositol was significantly reduced in the serum on days 21 and 35 pi in the present study, in contrast to findings by Oladosu et al. [41] showing increased myo-inositol in plasma of chickens with mixed gastrointestinal helminth infections, including ascarids, in weeks 2, 6, and 8 pi. This discrepancy most likely reflects different stages of the host’s reaction to infection rather than a contradiction. The lower levels observed in the present study might suggest active consumption or utilization of myo-inositol in sustained immune responses against parasitic infection, whereas the elevation seen by Oladosu et al. [41] might represent metabolic adaptation to support immune function. The significance of myo-inositol for immune barrier function is highlighted by the fact that myo-inositol deficiency hampered intestinal immune functions in grass carp [42]. Moreover, myo-inositol supplementation improved intestinal barrier integrity in vitro [43]. Thus, the reduced myo-inositol levels during *A. suum* infection might be a biomarker for chronic parasitic challenge in pigs and could reflect metabolic costs of preserving gut barrier function and developing long-lasting anti-helminth immunity.

## Conclusions

This study highlights the intricate interplay between host, parasite, and microbiome, revealing distinct roles of different intestinal compartments in responding to *A. suum* infection. Notably, the colon emerged as a key driver of systemic metabolic changes, particularly involving short-chain fatty acids and amino acids, which opens new avenues for targeted therapeutic strategies. Moreover, the observed metabolic recovery and enhanced growth in trickle-infected pigs suggest that chronic infections may trigger adaptive host responses with potential benefits. These insights not only deepen our understanding of *A. suum* pathophysiology in pigs, but also provide valuable perspectives for advancing research and treatment of human ascariaois. Altogether, the findings emphasize the importance of developing intervention approaches that consider the complex ecological dynamics of the gut to sustainably improve animal health and productivity.

## Supplementary Information

Additional file 1. Summary of all model parametersof PCA, OPLS-DA, or PLS-DA models

Additional file 2. Principal component analysis score scatter plot from ^1^H NMR spectrum profiles of jejunum from *Ascaris suum*-infected and uninfected control pigs.

## Data Availability

The metabolomics data have been deposited to MetaboLights [44] repository with the study identifier MTBLS13215.

## Abbreviations

CI: Confidence interval
CDCl3: Deuterated chloroform
CD3OD: Deuterated methanol
COSY: Correlation spectroscopy
CV-ANOVA: Cross validation analysis of variance
D2O: Deuterium oxide
DSS: 3-(Trimethylsilyl)-1-propanesulfonic acid-d6 sodium salt
HSQC: Heteronuclear single quantum correlation
NAC: N-acetylcysteine
NMR: Nuclear magnetic resonance
OPLS-DA: Orthogonal partial least squares-discriminant analysis
PCA: Principal component analysis
PLS-DA: Partial least squares-discriminant analysis
SD: Standard deviation
TCA: Tricarboxylic acid cycle
TOCSY: Total correlation spectroscopy
TSP: D4-sodium-3-(trimethylsilyl)-2,2,3,3-tetradeuteriopropionate
VIP: Variable influences in projection
