# Supplementary material for: 1H NMR-based metabolomics reveals metabolic changes in porcine ingesta and serum during Ascaris suum infection
Source: Parasit Vectors. 2026 May 23;19:229. doi: 10.1186/s13071-026-07423-z (PMC13198747; doi:10.1186/s13071-026-07423-z)
Supplement: Supplementary file 1 — Additional file 1. Summary of all model parametersof PCA, OPLS-DA, or PLS-DA models [file 13071_2026_7423_MOESM1_ESM.docx]

|  | **Samples^1^** | **Model^2^** | **Components^3^** | **R2X [%]^4^** | **Q2 [%]^5^** | **R2Y [%]^6^** | **CV-ANOVA^7^** | | **Figure** |
| --- | --- | --- | --- | --- | --- | --- | --- | --- | --- |
| **Ingesta** | all | PCA | 24 | 99.0 | 97.4 |  | |  | 3A & B |
|  |  | OPLS-DA | 2+0+0 | 82.3 | 1.75 | 3.41 | | 0.3124 |  |
|  |  | PLS-DA | 5 | 93.3 | 1.18 | 5.55 | | 1 |  |
| Jejunum | all | PCA | 17 | 98.6 | 95.0 |  | |  | S1 |
|  |  | OPLS-DA | 1+0+0 | 54.7 | -1.31 | 1.38 | | 1 |  |
| Ileum | all | PCA | 10 | 95.7 | 84.3 |  | |  | 4A |
|  |  | (O)PLS-DA | - |  |  |  | |  |  |
| quant^8^ | 35 pi | PCA | 4 | 62.7 | -8.07 |  | |  | 4B |
|  |  | OPLS-DA | 2+2+0 | 56.6 | 75.1 | 96.4 | | 0.0170 | 4C |
| Caecum | all | PCA | 8 | 90.5 | 73.7 |  | |  | 6A |
|  |  | OPLS-DA | 1+0+0 | 45.5 | -0.47 | 4.26 | | 1 |  |
| quant | 21 pi | PCA | 2 | 50.7 | 6.84 |  | |  | 6B |
|  |  | OPLS-DA | 1+1+0 | 34.2 | 0.95 | 40.9 | | 1 | 6C |
| quant | 35 pi | PCA | 3 | 66.0 | 16.7 |  | |  | 6D |
|  |  | OPLS-DA | 2+0+0 | 46.6 | 24.8 | 56.3 | | 0.7968 | 6E |
| Colon | all | PCA | 12 | 94.6 | 79.1 |  | |  | 7A |
|  |  | OPLS-DA | 2+0+0 | 58.5 | 0.41 | 11.5 | | 0.9894 |  |
| quant | 21 pi | PCA | 3 | 69.9 | 22.3 |  | |  | 7B |
|  |  | OPLS-DA | 1+0+0 | 32.5 | 0.64 | 28.8 | | 1 | 7C |
| quant | 35 pi | PCA | 2 | 53.8 | 22.8 |  | |  | 7D |
|  |  | OPLS-DA | 1+1+0 | 41.5 | 24.9 | 43.8 | | 0.9911 | 7E |
| quant | 49 pi | PCA | 3 | 59.4 | 12.6 |  | |  | 7F |
|  |  | OPLS-DA | 2+0+0 | 40.0 | 15.0 | 60.1 | | 0.9996 | 7G |
| **Serum** | all | PCA | 9 | 93.3 | 80.9 |  | |  | 8A |
|  |  | OPLS-DA | 2+0+0 | 65.8 | -0.16 | 0.83 | | 1 |  |
|  | 21 pi | PCA | 4 | 53.7 | -16.1 |  | |  | 8B |
|  |  | OPLS-DA | 1+1+0 | 28.5 | 21.3 | 50.2 | | 0.9586 | 8C |
|  | 35 pi | PCA | 3 | 62.7 | 15.8 |  | |  | 8D |
|  |  | OPLS-DA | 1+2+0 | 57.6 | 20.8 | 50.7 | | 0.9990 | 8E |
|  | 49 pi | PCA | 1 | 52.7 | 44.0 |  | |  | 8F |
|  |  | OPLS-DA | 2+2+0 | 70.9 | 17.6 | 84.3 | | 0.9999 | 8G |

**Additional file 1:** Summary of all model parameters (number of components, R^2^X, Q^2^, R^2^Y, CV-ANOVA) of PCA, OPLS-DA or PLS-DA models.

^1^samples: in regards of the different parts of the ingesta and serum the data analysis was conducted from all samples from the whole time period (all) or at the different days, e.g. at day 21 (21 pi), day 35 (35 pi) or day 49 (49 pi) post infection (pi)

^2^models: PCA (principal component analysis); OPLS-DA (orthogonal partial least squares projection to latent structures-discriminant analysis); PLS-DA (partial least squares projection to latent structures-discriminant analysis)

^3^components: that capture variation found in X and Y are donated predictive. OPLS-DA: variation only found in X or Y are denoted orthogonal in X(OPLS) or Y(OPLS), respectively (e.g. 1+1+0: one predictive and one orthogonal in X and zero orthogonal in Y)

^4^Cumulative fraction of X variation modeled in that component, using the X model

^5^Cumulative fraction of Y variation predicted by the X model in that component, according to cross-validation

^6^Cumulative fraction of the Y variation modeled in that component, using the Y model

^7^CV-ANOVA, ANalysis Of VAriance testing of Cross-Validated predictive residuals, is an analytical tool for judging the reliability of PLS and OPLS models

^8^quant: quantitative analysis (profiling approach, using absolute concentrations)
