# Supplementary figures and images for: 1H NMR-based metabolomics reveals metabolic changes in porcine ingesta and serum during Ascaris suum infection
Source: Parasit Vectors. 2026 May 23;19:229. doi: 10.1186/s13071-026-07423-z (PMC13198747; doi:10.1186/s13071-026-07423-z)

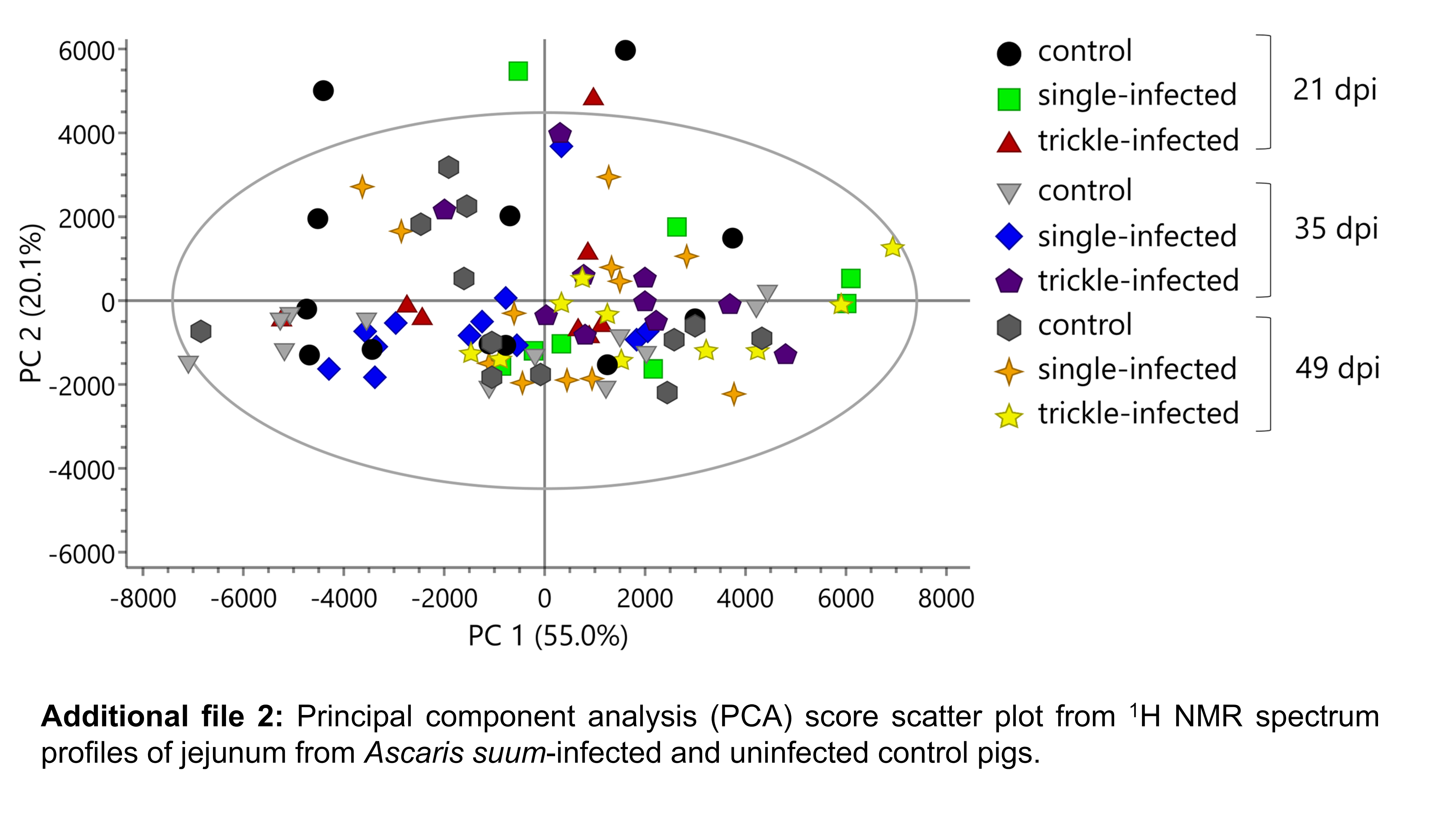

Supplement: Supplementary file 2 — Additional file 2. Principal component analysis score scatter plot from 1H NMR spectrum profiles of jejunum from Ascaris suum-infected and uninfected control pigs. [file 13071_2026_7423_MOESM2_ESM.tif]
